# Supplementary material for: Preliminary Study on Expression and Function of the Chicken W Chromosome Gene MIER3 in Embryonic Gonads
Source: Int J Mol Sci. 2023 May 17;24(10):8891. doi: 10.3390/ijms24108891 (PMC10218813; doi:10.3390/ijms24108891)
Supplement: Supplementary file 1 [file ijms-24-08891-s001.zip › Supplementary Information..pdf]

Supplementary Information

Table S1 Sequences of primers for qPCR.

Table S2 Down regulated genes in male gonadal cells transfected with *MIER3*-Pex2.

Table S3 Up regulated genes in male gonadal cells transfected with *MIER3*-Pex2.

Table S4 Differentially expressed genes after transcriptome sequencing (see Excel file).

Table S5 The list of GO terms significantly enriched with differentially expressed genes (see Excel file).

Table S6 The list of KEGG pathways significantly enriched with differentially expressed genes (see Excel file).

Figure S1 Some specific sequences in the protein sequence of chicken *MIER3* gene are different. ① is Figure a, ② is Figure b, and ③ is Figure c

Figure S2 Standard curves for detecting the expression level of chicken *MIER3* gene using fluorescence quantitative PCR method. The abscissa is a multiple of 2. *MIER3W*-127 refers to the design of primers for the same sequence of the first, second, and seventh transcripts of the MIER3-W gene. *MIER3W*-3, *MIER3W*-45, and *MIER3W*-6 are similar to *MIER3W*-127. *MIER3W* refers to the design of primers for the same sequence of the seven transcripts of the *MIER3* gene, and *HMBS* is an internal reference gene. **Figure S3** Expression levels of different transcripts of *MIER3* in the gonads of E12 chicken embryos. **Figure S4** Evolutionary tree of MIER3 protein.

Figure S5 Relative mRNA expression of chicken *MIER3-W*, *MIER3-Z* and *MIER3-ZW* in different embryonic tissues in E6 (n=5) and E12 (n=5). The mRNA expression levels were shown as values relative to that of female gonad. SPSS 16.0 (SPSS China, Shanghai, China) was used to perform the Student's t-test for statistical significance of differences between male and female tissues. There was significant difference among different alphabetic columns ($P < 0.05$), * and * * represented $P < 0.05$ and $P < 0.01$,

and the data were mean ± standard deviation.

Figure S6 The relative expression of *MIER3-Z* in the left gonad of chicken embryo was detected by qPCR. The mRNA expression levels were shown as values relative to that of female gonad. SPSS 16.0 (SPSS China, Shanghai, China) was used to perform the Student's t-test for statistical significance of differences between male and female tissues. * and * * represented $P < 0.05$ and $P < 0.01$.

Figure S7 Volcano map for differentially expressed genes. Note: The abscissa is log2(Fold Change), the ordinate is -log10 (p-value), and the blue dotted line indicates the threshold line of screening criteria for differentially expressed genes. The red and green dots indicate the genes with upregulated and downregulated expression in Chicken embryo male gonad cells transfected with *MIER3* overexpression vector vs. empty vector, respectively.

Figure S8 The histogram showing the results from GO enrichment analysis. Note: The coordination shows GO term on the abscissa and the significance of enrichment on the ordinate. The significance of enrichment is indicated by -log10 (padj). Padj denotes adjusted *P* value. Different colors represent different categories of GO term, including biological process (BP), cellular component (CC) and molecular function (MF).

Table S1 Primer sequences for quantitative PCR analysis

Gene	GenBank Number	Primer Sequence (5'-3')	Product Size
CCL4	NM_204720.3	F:CCTGCAGACTAGAAAGGCCC<br>R:AAATGCGCTCCCTACCACTC	135
CDKN2A	NM_204434.1	F:GCGGAAGAAGACACCGCA<br>R:GAATACGGCGAGCATCGGA	235
CDKN2B	NM_204433.2	F:GCGGGGATTAGAGGGATGTG<br>R:TCGGACCGAGGCTTATCAGA	181
CHAC1	NM_001199656.2	F:TCCGGTGCAATACAAAGTGA<br>R:ACCAATGCGAAGGTGAGC	190
CPLX1	XM_046905843.1	F:GAGCCATCGCCATGGATTTT<br>R:TTGGCAGCATCAGGGTCTTT	106
EGR1	NM_204136.3	F:AACACGGCTACCGCCAATTA<br>R:CGGACTGTTCTCACTGGCTT	217
FREM1	XM_004949102.5	F:TTATCATAGGCAGCCCGAAAG<br>M:GGCGACAGTGGGAAAGACG	174
HMBS	XM_025143289.3	F:GCTTTAGCGGTGGAGGTTCG<br>R:TGCAACAGGGACACTACAGC	144
MIER3-W	XM_015300440.3	F:AGGTAGACAGTGGTAATACATCCG<br>R:TGTAGTCCTTCAGGAATTCTGCC	244
MIER3W-127		F:CAGGTTTATACCGCCTCCAC<br>R:TGCGGCCTAGTCCTGCAGACACCTG	96
MIER3W-3		F:GAGGCACCTTGTTCCAGACTA<br>R:TTCCATCATTTCCTCCTCTTCG	178
MIER3W-45		F:TGCTAGCGCTGTTGCTCAACC<br>R:CCTGCCATAACTGGAATCGTG	289
MIER3W-6		F:GAAGGAGCACTGGGTCAGTC<br>R:TCGAACTCCCAAAGGATGCC	125
MIER3-Z	XM_004937251.5	F:TCTCAAAGGGAGTACCGTGTG<br>R:ATGCAACGATAGCGACCTCC	100
MIER3-ZW		F:AGGAAACATGCCGTTGGAAGA<br>R:TCTGGAAGTTCATCTGCAAGT	117
PTGS2	NM_001167718.2	F:GACTCCGAAGTCAGTGCAGC<br>R:CGAGTTCCACTTGCACGAGA	145
RGS1	XM_422203.8	F:GGAAAGGAGGACTGCAAGCT<br>R:AGGAACTCCCGAAAGACACC	233
TNFRSF13B	NM_001097537.1	F:CACCAGGACAGGCAGACAAA<br>R:CCGATGGAGGAACTGGGAT	190
αGSU	NM_001278021.1	F:ATGCAGGGTTGTCCAGAGTG<br>R:AGCACGTTGCTTCCGATGTA	169
β-Actin	NM_205518.2	F:TATGTGCAAGGCCGGTTTC<br>R:TGTCTTTCTGGCCCATACCAA<br>R:TCGAACTCCCAAAGGATGCC	110

Table S2 Down regulated genes in gonadal cells transfected with *MIER3*-Pex2

**Name**	**Description**	***p*-Value**	**Fold Change**
PTGDS	prostaglandin D2 synthase 21kDa	0.0032	0.29
TGM6	transglutaminase 6	0.0032	0.32
NSUN7	NOP2/Sun RNA methyltransferase family member 7	0.0061	0.48
USHBP1	USH1 protein network component harmonin binding protein 1	0.0084	0.09
EGR1	early growth response 1	0.0111	0.32
HAL	histidine ammonia-lyase	0.0138	0.46
FREM1	FRAS1 related extracellular matrix 1	0.0145	0.39
MSMP	microseminoprotein, prostate associated	0.0165	0.14
TNFRSF13B	TNF receptor superfamily member 13B	0.0249	0.34
TNFRSF18	TNF receptor superfamily member 18	0.0428	0.47
CAMK2A	calcium/calmodulin dependent protein kinase II alpha	0.0474	0.35
ACOT12	acyl-CoA thioesterase 12	0.0495	0.49

Table S3 Up regulated genes in gonadal cells transfected with *MIER3*-Pex2

**Name**	**Description**	***p*-Value**	**Fold Change**
CPLX1	complexin 1	< 0.001	1.68
CCL4	C-C motif chemokine ligand 4	< 0.001	2.7
PTGS2	prostaglandin-endoperoxide synthase 2	< 0.001	1.99
CDKN2A	cyclin-dependent kinase inhibitor 2A (melanoma, p16, inhibits CDK4)	< 0.001	1.91
CDKN2B	cyclin dependent kinase inhibitor 2B	< 0.001	1.85
PCASP2	paracaspase 2	0.0011	11.51
CSF3	colony stimulating factor 3	0.0105	2.04
NFASC	neurofascin	0.0145	2.26
DCAF11	DDB1 and CUL4 associated factor 11	0.0197	5.18
CDH5	cadherin 5	0.0232	2.07
EN2	engrailed homeobox 2	0.0374	9.11
HSPB3	heat shock protein family B (small) member 3	0.0464	2.33

Figure S1

A

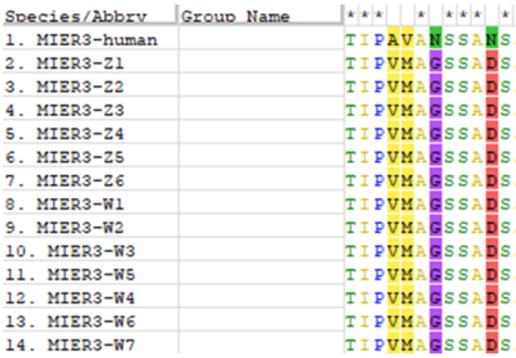

B

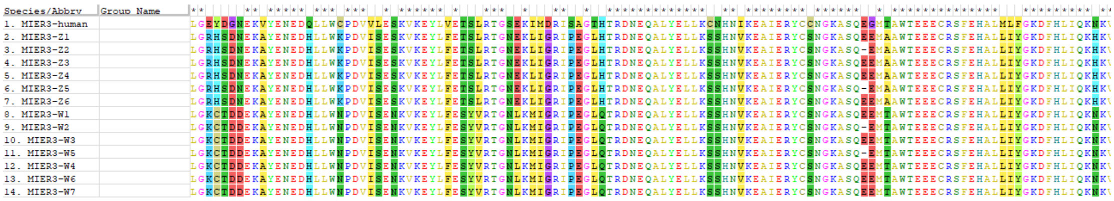

C

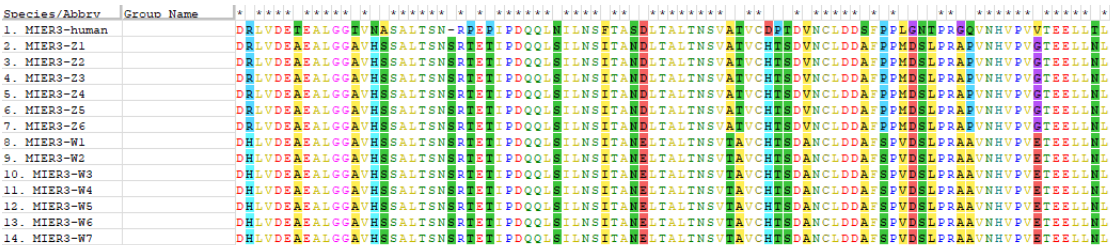

Figure S2

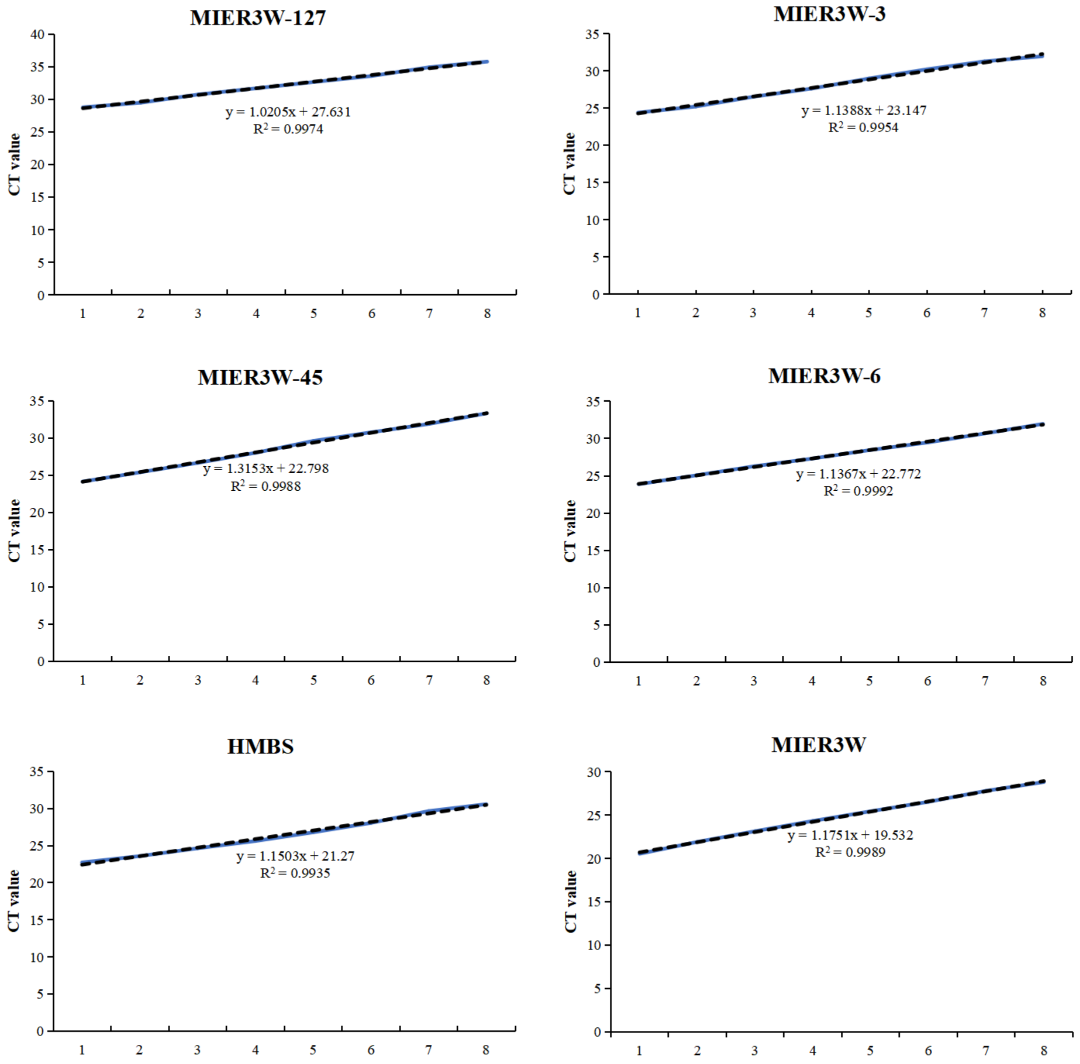
MIER3W-127
y = 1.0205x + 27.631
R² = 0.9974
CT value
MIER3W-3
y = 1.1388x + 23.147
R² = 0.9954
CT value
MIER3W-45
y = 1.3153x + 22.798
R² = 0.9988
CT value
MIER3W-6
y = 1.1367x + 22.772
R² = 0.9992
CT value
HMBS
y = 1.1503x + 21.27
R² = 0.9935
CT value
MIER3W
y = 1.1751x + 19.532
R² = 0.9989
CT value

Figure S3

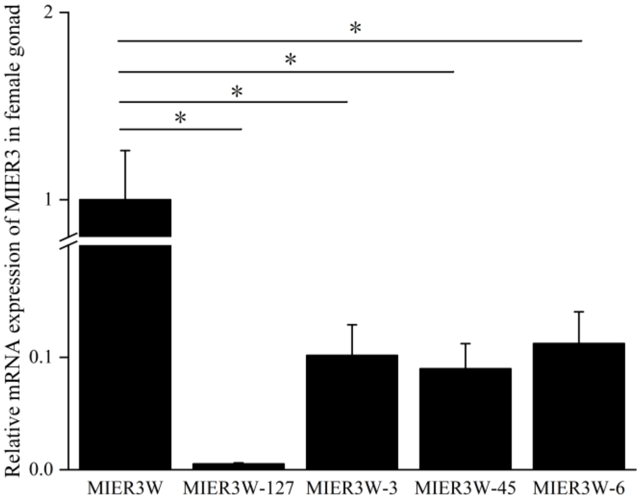
Relative mRNA expression of MIER3 in female gonad
2
1
0.1
0.0
*
*
*
*
MIER3W
MIER3W-127
MIER3W-3
MIER3W-45
MIER3W-6

Figure S4

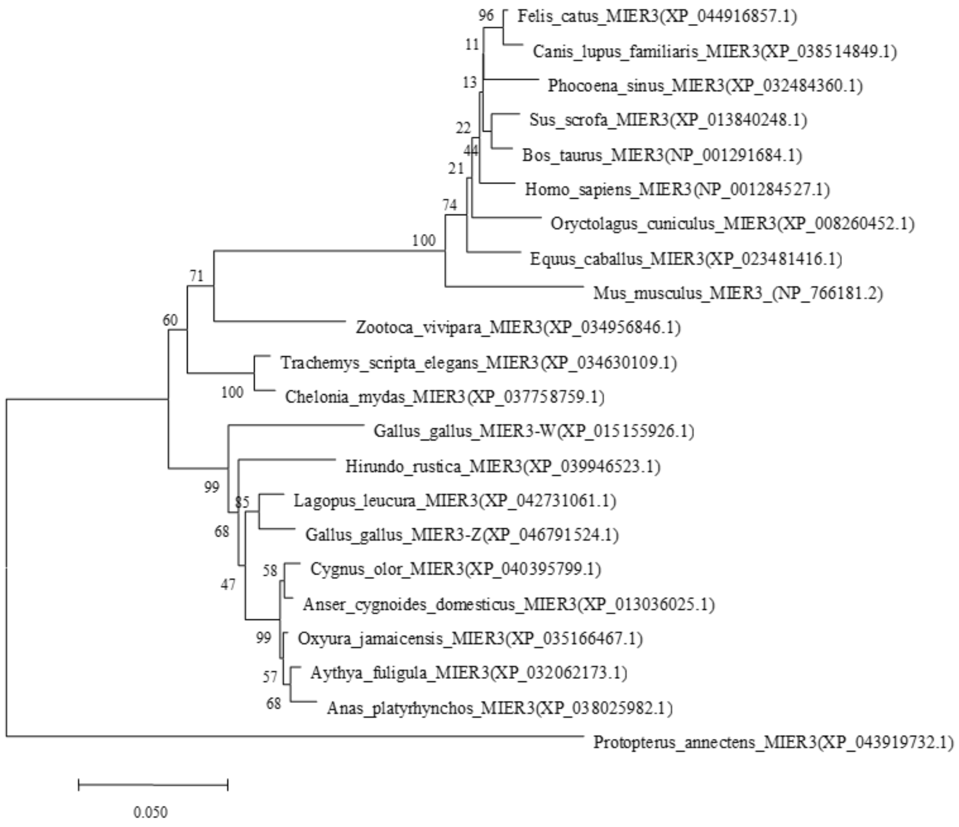

Felis_catus_MIER3(XP_044916857.1)
Canis_lupus_familiaris_MIER3(XP_038514849.1)
Phocoena_sinus_MIER3(XP_032484360.1)
Sus_scrofa_MIER3(XP_013840248.1)
Bos_taurus_MIER3(NP_001291684.1)
Homo_sapiens_MIER3(NP_001284527.1)
Oryctolagus_cuniculus_MIER3(XP_008260452.1)
Equus_caballus_MIER3(XP_023481416.1)
Mus_musculus_MIER3_(NP_766181.2)
Zootoca_vivipara_MIER3(XP_034956846.1)
Trachemys_scripta_elegans_MIER3(XP_034630109.1)
Chelonia_mydas_MIER3(XP_037758759.1)
Gallus_gallus_MIER3-W(XP_015155926.1)
Hirundo_rustica_MIER3(XP_039946523.1)
Lagopus_leucura_MIER3(XP_042731061.1)
Gallus_gallus_MIER3-Z(XP_046791524.1)
Cygnus_olor_MIER3(XP_040395799.1)
Anser_cygnoides_domesticus_MIER3(XP_013036025.1)
Oxyura_jamaicensis_MIER3(XP_035166467.1)
Aythya_fuligula_MIER3(XP_032062173.1)
Anas_platyrhynchos_MIER3(XP_038025982.1)
Protopterus_annectens_MIER3(XP_043919732.1)
96
11
13
22
44
21
74
100
71
60
100
99
85
68
58
47
99
57
68
0.050

Figure S5

A

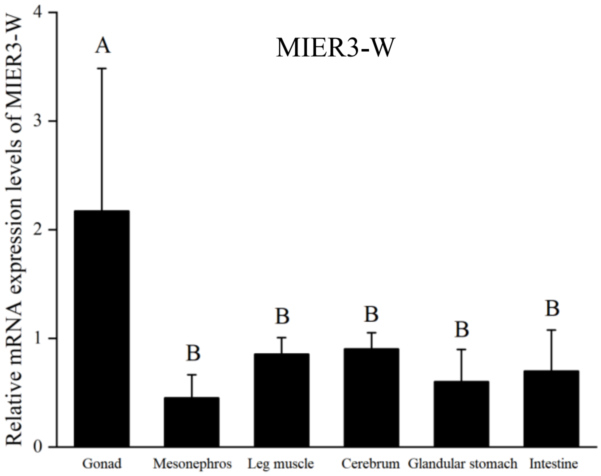

B

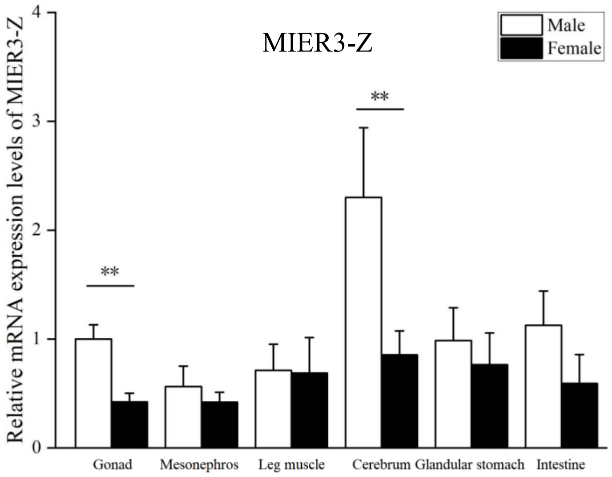

C

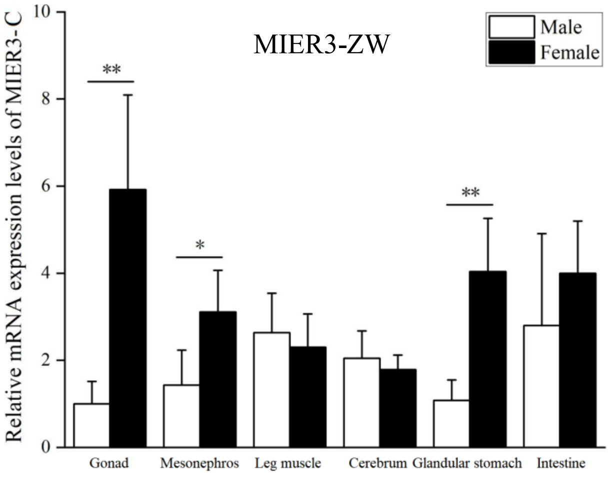

D

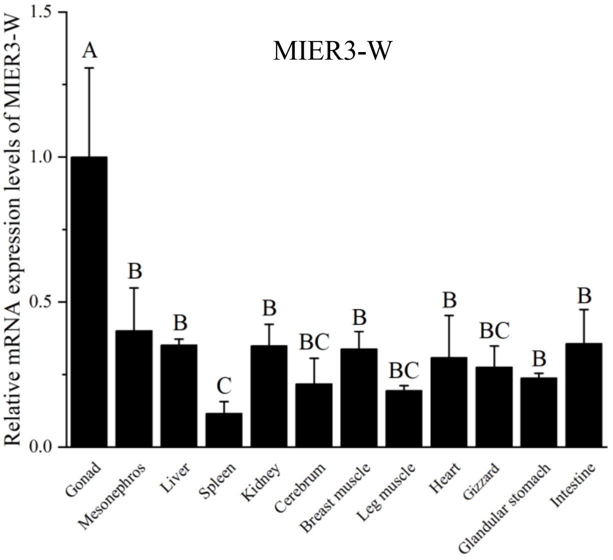
MIER3-W
Relative mRNA expression levels of MIER3-W
1.5
1.0
0.5
0.0
A
B
B
C
B
BC
B
BC
B
BC
B
B
Gonad
Mesonephros
Liver
Spleen
Kidney
Cerebrum
Breast muscle
Leg muscle
Heart
Gizzard
Glandular stomach
Intestine

E

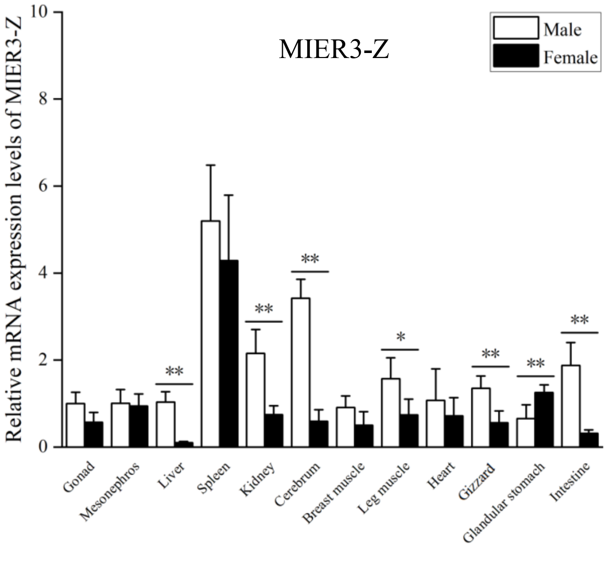
MIER3-Z
Male
Female
Relative mRNA expression levels of MIER3-Z
10
8
6
4
2
0
**
**
**
*
**
**
**
Gonad
Mesonephros
Liver
Spleen
Kidney
Cerebrum
Breast muscle
Leg muscle
Heart
Gizzard
Glandular stomach
Intestine

F

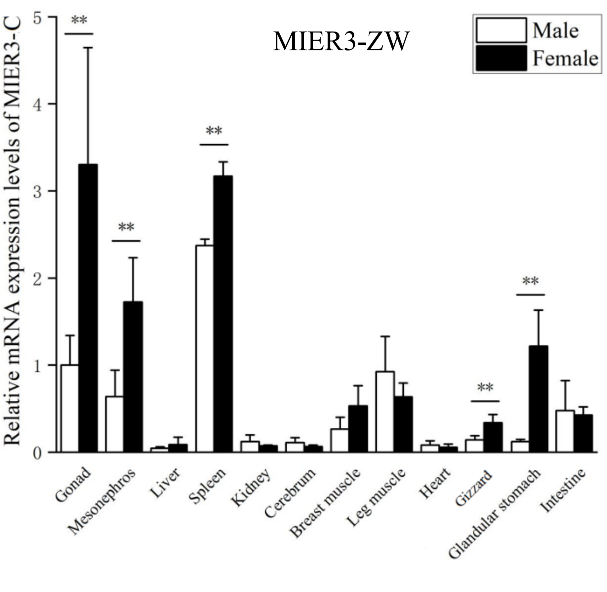
MIER3-ZW
Male
Female
Relative mRNA expression levels of MIER3-C
5
4
3
2
1
0
**
**
**
**
**
Gonad
Mesonephros
Liver
Spleen
Kidney
Cerebrum
Breast muscle
Leg muscle
Heart
Gizzard
Glandular stomach
Intestine

Figure S6

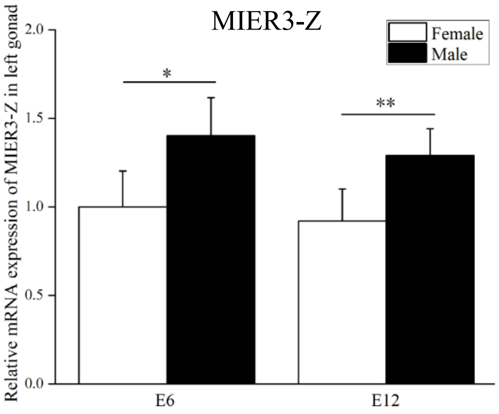
MIER3-Z
Female
Male
Relative mRNA expression of MIER3-Z in left gonad
2.0
1.5
1.0
0.5
0.0
*
**
E6
E12

Figure S7

.

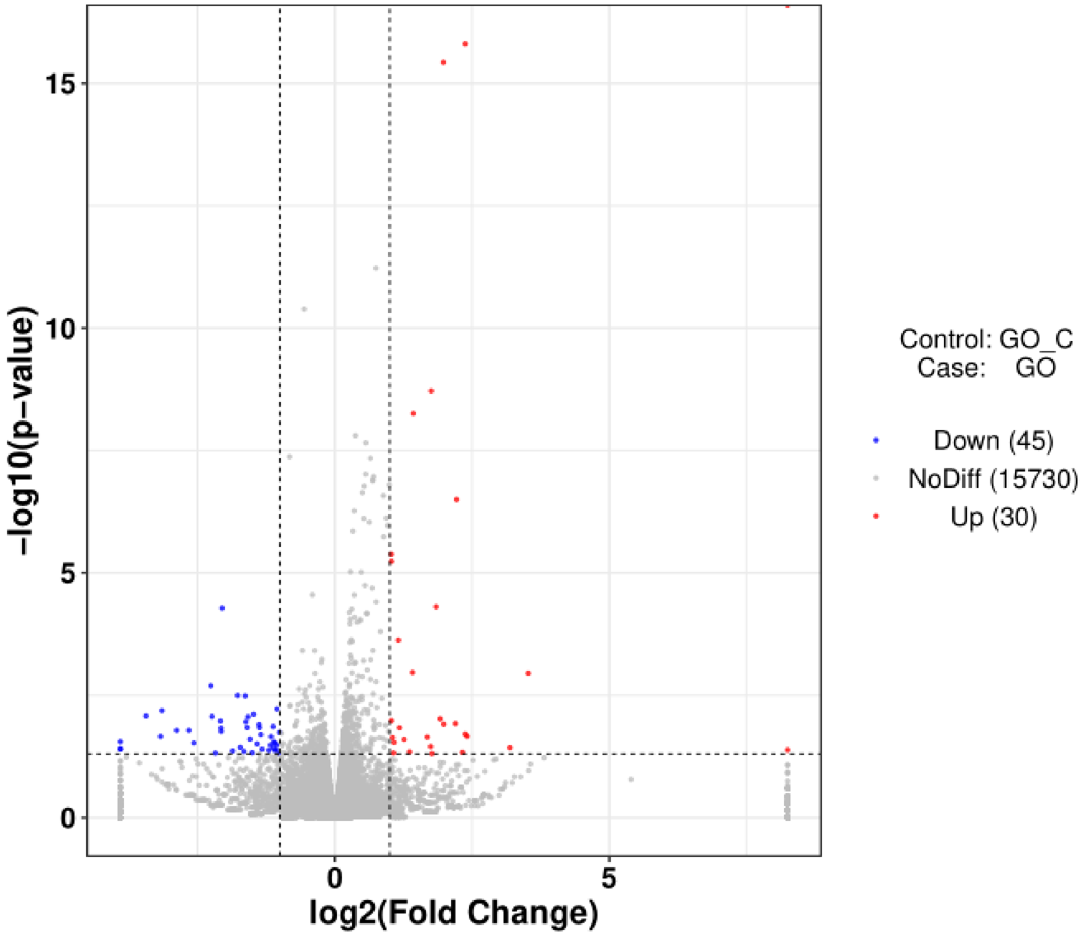

Note: The abscissa is log2(Fold Change), the ordinate is -log10(*P*-value), and the blue dotted line indicates the threshold line of screening criteria for differentially expressed genes. The red and green dots indicate the genes with upregulated and downregulated expression in Chicken embryo male gonad cells transfected with *MIER3* overexpression vector vs. empty vector, respectively.

Figure S8

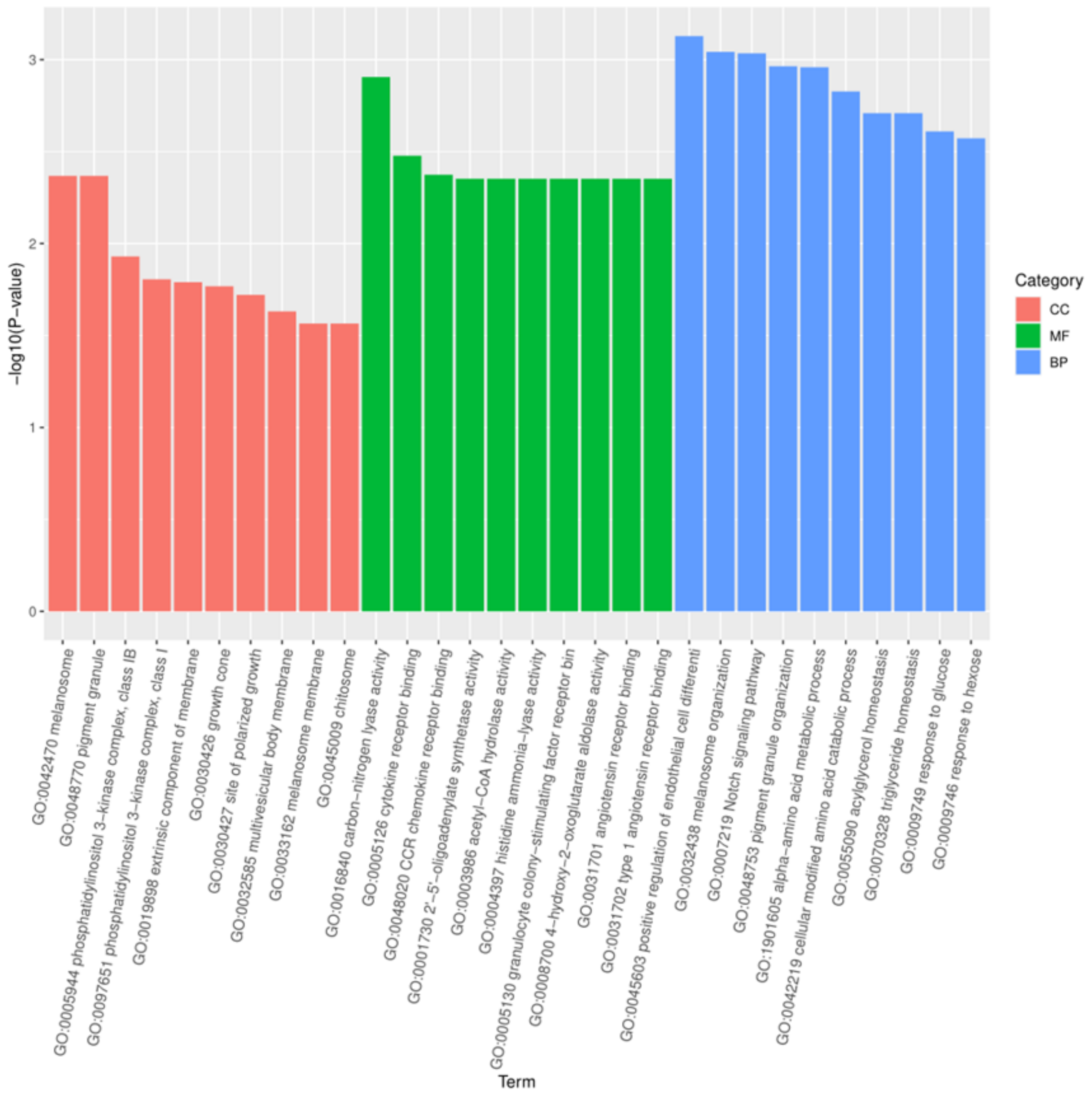

Note: The coordination shows GO term on the abscissa and the significance of enrichment on the ordinate. The significance of enrichment is indicated by -log10 (padj). Padj denotes adjusted p value. Different colors represent different categories of GO term, including biological process (BP), cellular component (CC) and molecular function (MF).
